# Digital Presence of a Research Center as a Research Dissemination Platform: Reach and Resources

**DOI:** 10.2196/11686

**Published:** 2019-04-05

**Authors:** Sarah E Lord, Katherine M Seavey, Sonia D Oren, Alan J Budney, Lisa A Marsch

**Affiliations:** 1 Center for Technology and Behavioral Health Geisel School of Medicine Dartmouth College Lebanon, NH United States

**Keywords:** telemedicine, internet, social media, behavioral sciences, implementation science, information dissemination

## Abstract

**Background:**

Web-based platforms can be powerful tools for research dissemination. By leveraging the advantages of mass media and interpersonal channels of communication, Web-based dissemination platforms may improve awareness about, and subsequent adoption of, evidence-based practices (EBPs). Digital dissemination strategies can augment traditional dissemination models, improving stakeholder access to digestible and actionable information and promoting translation of EBPs.

**Objective:**

This study aimed to describe the reach and content of the Web presence of a National Institute on Drug Abuse Center of Excellence and how it is used to disseminate research related to digital behavioral health approaches.

**Methods:**

The Center for Technology and Behavioral Health (CTBH) has a website and regularly updated Facebook and Twitter accounts. The website features include summaries of digital behavioral health approaches and related empirical literature, a blog feed focused on the state of the science and technology concerning digital health care approaches, and a newsletter about Center activities. We extracted website usage metrics from Google Analytics and follower counts from social media accounts for the period from March 1, 2013, to July 17, 2018.

**Results:**

Since the implementation of analytic tracking, 70,331 users have initiated 96,995 sessions on the CTBH website. The website includes summaries of 86 digital therapeutic programs, encompassing 447 empirical articles. There are 1160 posts in the CTBH blog feed, including 180 summaries of scholarly articles. The Twitter and Facebook accounts have 577 and 1500 followers, respectively. The newsletter has reached a growing subscriber network and has a high open rate relative to industry standards.

**Conclusions:**

The CTBH Web presence serves as a model for how to leverage accessible and easily updatable digital platforms as research dissemination channels. Digital dissemination tools can augment traditional dissemination strategies to promote awareness about evidence-based digital therapeutic approaches for behavioral health and health care more broadly.

## Introduction

### Background

The limited translation of research to practice is a long-standing issue in health care [1-3], and adoption of innovations to support evidence-based treatment for substance use and mental health conditions is no exception. Barriers to translation include limited stakeholder awareness of evidence-based practices (EBP) and their demonstrated impact for target patient populations as well as clinician expectations that adoption of new practices may increase burden [4-6]. Strategies to make communication of research findings more relevant, digestible, and actionable to clinician and policy stakeholders may improve dissemination and adoption of empirically supported practices [7].

### The Promise of Digital Treatment Approaches to Substance Use and Mental Health Care

Technology offers great promise for addressing many of the barriers to adoption of EBP [5,8]. Digital behavioral health approaches, such as Web-based programs, mobile apps, and text messaging, can transcend the time and geographic boundaries of traditional clinical practice settings and offer evidence-based care to broad patient audiences when and where they need it. Different mixed media (eg, text, audio, and video) can be used in digital therapeutic approaches to appeal to a broad range of learning styles [9] and promote engagement [10,11]. There is strong and growing evidence for digital therapeutic approaches targeting substance use and mental health conditions [12-17]. These digital behavioral health approaches have demonstrated efficacy for identifying symptoms [18,19], evoking positive behavior change [20-28], and facilitating recovery support [29]. Despite a promising impact, digital therapeutic approaches for behavioral health have yet to see widespread implementation in practice.

### Technology as a Platform for Research Dissemination

Technology—and, in particular, the internet—can also serve as a powerful platform for science dissemination to patient, provider, researcher, and policy stakeholders. Digital platforms can bridge approaches to dissemination and diffusion of research, allowing information to reach broad audiences with little effort required from the researcher beyond posting the information. According to the diffusion of innovations theory [30], key dissemination channels include interpersonal and mass media channels of communication. Mass media channels of communication are the most efficient, but interpersonal communication channels are the most persuasive [30]. Web platforms may be able to leverage the strengths of both channels of communication. There is preliminary evidence that Web platforms share features of mass media (eg, reaching many people at once) and interpersonal channels of communication (eg, the ability to cultivate homophilous communities) [31]. Web-based platforms may fall into an entirely new channel of communication: *new media*, which shares the efficiency of mass media and personalization of interpersonal communication [32].

Studies have highlighted the internet, including social media, as a useful tool for researchers to disseminate information about EBPs to the public [33-36]. Websites related to research, such as blogs, can have higher traffic than Web-based journal articles about comparable topics [37]. Through digital media, research findings can be disseminated to broad audiences using language that is relevant and accessible, user-friendly, and integrated with social media to promote communication [34-36]. Such Web-based dissemination efforts can complement and extend dissemination of research via scientific publications; presentations to scientific, policy, and community audiences; and mass media coverage. To our knowledge, only 1 other study has described how an academic institutional website was used as a tool for research dissemination—in this case, focused on implementation of shared decision making in primary care [38].

Current initiatives by the National Institute on Drug Abuse (NIDA), the Substance Abuse and Mental Health Services Administration, and the Center for Substance Abuse Treatment focus on closing the science-to-practice gap through greater attention to the promotion of successful dissemination and implementation strategies for EBPs [39-41]. In this paper, we describe how website and social media features of a NIDA-designated P30 *Center of Excellence* (the Center for Technology and Behavioral Health [CTBH]) are used to disseminate research about digital therapeutic approaches for substance use and mental health conditions. Established in 2011, CTBH is a national and international leader in research focused on development, evaluation, dissemination, and implementation of digital behavioral health treatment approaches. CTBH maintains a website [42] as well as social media accounts to foster dissemination of research focused on digital behavioral health approaches and to promote awareness of CTBH activities and resources. The publicly available CTBH website and Facebook and Twitter accounts (collectively, CTBH Web presence) have the potential to reach broad audiences, including patients, clinicians, researchers, and policy makers.

Leveraging digital platforms for research knowledge translation has received little attention in translational research. Digital platforms can extend the reach of scientific knowledge to stakeholders who may not read academic journals [43]. Digital dissemination channels can allow for ready access to continuously updated information. Abundant and current content on a website promotes audience engagement [44]. Furthermore, posting about research on social media (eg, Twitter) facilitates uptake of research [37,45,46]. These digital channels are a key part of the mission of CTBH to be a national and international resource underscoring science-based digital behavioral health. The purpose of this paper was to present a model for leveraging digital platforms as research dissemination tools by describing CTBH’s Web presence and presenting data about its content and reach.

## Methods

### Center for Technology and Behavioral Health Web Presence

CTBH maintains a center-specific website and Twitter and Facebook accounts to keep users informed about the state of the science regarding digital behavioral health approaches and CTBH activities. CTBH launched its website in May 2012. Screenshots of the website features are available in Multimedia Appendix 1. What follows is a brief description of the website content.

#### The Center

*The Center* is the gateway to pages listing CTBH leadership, members and affiliates, publications and presentations, active projects, hosted seminars, and announcements (eg, recruitment for research studies) and news stories about CTBH activities. Users can view and subscribe to the CTBH newsletter from this page.

#### Program Review

The *Program Review* is a filterable compendium of annotated summaries of empirical literature for specific digital approaches for the treatment of substance use disorders, co-occurring disorders, mental health conditions, and HIV. Programs are identified for review through scoping literature searches of academic databases for digital interventions targeting the aforementioned conditions. Literature for each program review is extracted from the searches, lists of works by the investigators, and bibliographies of relevant articles.

Program reviews include descriptions of each intervention and information about the target populations with which each intervention has been evaluated (eg, age, race, and gender), delivery method (eg, Web-based or mobile app), guiding theoretical models (eg, community reinforcement approach, cognitive behavior therapy, or self-regulation theory), intervention target (eg, substance use or depression), outcomes (eg, reduced substance use, abstinence, symptom severity, or treatment adherence), and languages and countries in which the intervention has been delivered and evaluated. Each program review includes summaries of the studies evaluating the digital therapeutic tool, including methodology, results, and implications, and citations for nonevaluation articles related to the program (eg, study protocols). The key characteristics identified in program reviews are associated with fostering successful implementation [47] and align with recent recommendations for standardization of reporting on digital therapeutic tools [48,49]. Users can filter programs by target age group, target substance or condition, type of program, and commercial availability. Program review summaries are updated monthly.

#### Eye on Innovation

*Eye on Innovation* is a blog-style newsfeed that includes links to, and excerpts from, news stories relating to recent developments in digital health and an annotated bibliography of recently published empirical literature related to emerging technology and health (*Cutting Edge Literature*). Content features are tagged to allow for easy filtering by users. The newsfeed is updated weekly.

#### Social Media and Newsletter

CTBH launched its Twitter account in September 2013 and Facebook account in August 2014. These accounts feature posts about news related to technology in behavioral health, innovative research, and updates about CTBH activities. The Twitter and Facebook accounts are updated at least daily. CTBH distributes a quarterly newsletter by email to a subscriber distribution list. Subscribers can join this list from the CTBH website or by signing up at CTBH-hosted events.

### Sources of Data

We used Google Analytics to collect data on CTBH website usage. Information about social media audience is publicly displayed on the Facebook and Twitter accounts. We used information available from distribution service (MailChimp) reports to describe the audience and engagement with the newsletter over time.

### Data Extraction

CTBH began using Google Analytics to track user activity on the website in March 2013. For this report, we extracted data from March 1, 2013, to July 17, 2018, on the number of users, number of sessions initiated, number of page views, percentage of sessions initiated by new users, average number of pages viewed per session, average time spent on the site per session, most viewed pages, and countries where users were located. Information about numbers of Twitter and Facebook followers and posts was extracted from the CTBH Facebook and Twitter accounts. We used newsletter reports to extract data about the number of people who received and opened the first (April 2012) and most recent (December 2017) electronic newsletters and about the number of newsletter subscribers over time.

## Results

### Website Metrics

Between March 1, 2013, and July 17, 2018, 70,331 users initiated 96,995 sessions on the CTBH website. About 72.50% (70,317/96,995) of the sessions were initiated by new users. On average, users viewed about 2.4 pages per session and spent 1 min and 57 seconds on the site per session. Annual website usage metrics are available in Table 1. The most popular pages were the homepage (29,113 views), The Center (11,644 views), and the Program Review landing pages (10,826 views; Table 2). Users most often viewed the website from the United States (80.2%). Approximately 20% of the users were based in other countries (Table 3).

**Table 1:** Usage statistics for the Center for Technology and Behavioral Health website by year (March 1, 2013, to February 28, 2018).

Year^a^	Visits	Users	Page views	New user-initiated sessions, n (%)	Average pages per session	Average time per session
2013-2014	9365	6364	34,230	6297 (67.24)	3.7	2:57
2014-2015	17,383	12,379	48,035	12,198 (70.17)	2.8	2:12
2015-2016	17,534	13,156	46,697	12,959 (73.91)	2.7	1:57
2016-2017	17,062	12,130	52,779	11,983 (70.23)	3.1	2:35
2017-2018	25,288	19,173	39,881	19,008 (75.17)	1.6	1:18
Total (2013-2018)	86,632	63,505	221,622	62,445 (72.08)	2.6	2:03

^a^Years begin on March 1st and end on the final day of February of the following year.

**Table 2:** Most popular pages on the Center for Technology and Behavioral Health website based on page views (March 1, 2013, to July 17, 2018).

Page	Views (n)
Home	29,113
The Center	11,644
Program Review	10,826
Center Director	9654
Center Members	9465
Active Projects	6317
Eye on Innovation	5906
Center Leadership	5647
Programs for Substance Use Disorders	4354
Center Highlights	4257

**Table 3:** Most common locations from which users access the Center for Technology and Behavioral Health website: number and percentage of sessions (March 1, 2013, to July 17, 2018).

Country	Sessions, n (%)
United States	77,823 (80.23)
Canada	2514 (2.59)
United Kingdom	2026 (2.09)
India	1589 (1.64)
Australia	947 (0.98)

### Program Review

As of July 17, 2018, research related to 86 digital behavioral health therapeutic approaches were included in the Program Review. Within the 86 program reviews, there are 308 summaries of empirical studies evaluating the different digital behavioral health approaches and citations for 139 related articles (eg, secondary analyses). The most frequently viewed program review was Therapeutic Education System [21], a Web-based intervention for substance use based on the community reinforcement approach (4337 page views).

### Eye on Innovation and Cutting Edge Literature

As of July 17, 2018, the Eye on Innovation feed contained 1160 posts, including 180 Cutting Edge Literature pieces. Tags for individual posts are extracted from a total of 171 tags. The most commonly used tags are mHealth (ie, an abbreviation for mobile health; 326 posts), mobile apps (262 posts), mental health (140 posts), substance use (129 posts), and policy and regulation (107 posts). The most frequently viewed post describes the Mobile App Rating Scale [50] (202 page views).

### Social Media and Newsletter

CTBH has made 4849 tweets on the CTBH Twitter account since its creation. The Twitter account has 577 followers. As of July 17, 2018, there were 701 posts on the CTBH Facebook account. The Facebook page has been liked 1478 times and has 1500 followers.

The first newsletter in April 2012 was sent to 337 subscribers and was opened by 21% of the recipients. The most recent December 2017 newsletter was sent to 1132 subscribers and was opened by 34% of the recipients. The number of newsletter subscribers has increased each year since it has been recorded.

## Discussion

### The Center for Technology and Behavioral Health Web Presence as a Dissemination Strategy

The usage statistics associated with the CTBH Web presence indicate that the digital dissemination strategy was effective in reaching large audiences with research information related to digital therapeutics for behavioral health. Although users were primarily from the United States, about a quarter of users were based internationally. Although we were not able to determine user characteristics in this study, anecdotal evidence from email inquiries from the CTBH website from an array of stakeholders, including researchers, policy makers, clinicians, and patient consumers, suggests that we were able to reach diverse user audiences. To our knowledge, the only other study describing the reach of a Web-based research dissemination platform saw an average of 7906 visits by 5382 users each year [38]. The CTBH Web presence sees thousands of users and visits each year and has seen a growth in audience since its inception, suggesting a high and expanding reach of this digital dissemination strategy.

In addition, the CTBH website shows evidence of *stickiness*, commonly defined as a user’s willingness to use and continue using a website [51,52]. An important metric of website stickiness is session duration. According to the Nielsen Group, users typically spend less than 1 min on a Web page, on average [53]. The average session duration on the CTBH website was 1 min and 57 seconds, indicating higher than average levels of stickiness. The CTBH newsletter has also seen a growing number of subscribers since its launch and has been consistently opened by a high percentage of subscribers. The open rate of the most recent newsletter (34%) is impressive compared with the first newsletter in April 2012 (21%) and with the industry average open rate (Education and Training: 22%) [54]. The CTBH Twitter and Facebook accounts provide hundreds of followers with daily updates about digital behavioral health research.

The CTBH Web presence can serve as a model for research dissemination. Anecdotally, we have heard from researchers that they wish they could use their website more effectively for research dissemination. We present several features of the CTBH Web presence as a demonstration of the variety of ways websites and social media can be effectively leveraged to disseminate research. We also present the quantity of content on the CTBH website to illustrate the abundance of updated content continuously available to users.

Though the content on a website is not necessarily indicative of successful dissemination, user engagement is related to websites having enough content to attract repeat visitors and having current information [44]. Similarly, posts on social media are not necessarily indicative of successful research dissemination, but early evidence suggests that social media promotion or discussion of research may promote engagement with research. Notably, articles that were frequently linked in tweets were found to be 11 times more likely to be highly cited in academic publications than articles that were less frequently mentioned in tweets [45]. Similarly, Valdez Soto et al [46] identified a potential link between social media promotion and enrollment in Web-based training, and Hoang et al [37] found that above-average activity on Web-based articles coincided with social media promotion, though increases in engagement in either study could not be concretely linked to social media promotion.

The use of multiple digital dissemination channels (eg, website, social media, and electronic newsletter) provides users with greater flexibility in how they access information about the science of digital behavioral health therapeutic approaches. End users may choose to check the website for updates, receive quarterly newsletters, or follow CTBH social media accounts for daily updates. The growing audiences on the website and followers on social media accounts demonstrate the success of the model for promoting reach and awareness about research related to digital therapeutic approaches. We hope that by describing the features of the CTBH Web presence, we can help other researchers to optimize their own Web presence for research dissemination to attract and engage user audiences.

### Limitations and Future Directions

One limitation of this study is that we did not conduct statistical analyses comparing website use with users’ actual use of evidence-based digital behavioral health approaches in practice or research. Evaluating the impact of this research dissemination model on user behavior is an important direction for future research. Another limitation is that we could not examine the early growth of website usage because Google Analytics was not implemented for the site until the year after its launch. In addition, usage data from Google Analytics did not allow us to get a detailed picture of those who viewed the website, including professions of users and whether users were affiliated with CTBH or from outside of the CTBH network. Future features of the Web presence will include metrics to assess user characteristics.

The data extracted from the CTBH social media accounts only offer a current picture of user interaction with the accounts; we were unable to extract historical usage data for the accounts. Finally, it is also relevant to mention that spam accounts on social media, although relatively infrequent, can distort user interaction statistics [55]. It may be difficult to identify spam accounts from a list of followers. Although some spam account behavior is markedly different from the behavior of legitimate accounts, other spam accounts display patterns of interaction similar to the behavior of legitimate accounts [56].

Future directions for the CTBH website as a research dissemination platform include expansion of existing features and addition of features to encourage interaction among users of the CTBH Web presence components. A *Resources* page of the website was introduced in 2017, which includes a developing compendium of measures for assessing implementation of digital therapeutic interventions across the stages of treatment development research continuum [57] and roadmaps for implementation of digital behavioral health therapeutic approaches in different care settings. Future resources will include Web-based consent protocols and best practice methods for research study participant recruitment on social media. CTBH plans to engage its audience through social media to promote conversation among user stakeholders about interests and experiences with digital behavioral health approaches, with the goal of fostering collaborations for future implementation studies.

CTBH aims to continue to disseminate timely information about the state of digital approaches to behavioral health and will further explore the potential of social media to personalize the digital dissemination experience. There is limited research on the role of new media as facilitators in the translation of research to practice. We have demonstrated that our digital dissemination strategy has achieved a good reach to end user stakeholders compared with other published work [38]. We plan to investigate the persuasiveness of this approach for promoting the use and adoption of digital therapeutics in future work.

### Conclusions

In this paper, we demonstrated the potential of digital strategies to successfully disseminate information about research related to digital behavioral health therapeutic approaches. Although Web presence can promote awareness about existing research, successful translation depends on the application of knowledge and implementation skills to promote the use of these digital approaches by patients-clients, providers, and researchers [58]. In future research, we will examine how stakeholders utilize information from the CTBH digital dissemination platforms. We will also explore strategies for using these platforms to promote collaboration among stakeholder groups to promote research agendas focused on the successful implementation of digital behavioral health approaches in diverse settings.

## Appendix

Multimedia Appendix 1 Screenshots of the Center for Technology and Behavioral Health Web presence features. Images were extracted on July 23, 2018.

## Abbreviations

CTBH: The Center for Technology and Behavioral Health
EBP: evidence-based practice
NIDA: The National Institute on Drug Abuse
