# Supplementary material for: Digital Presence of a Research Center as a Research Dissemination Platform: Reach and Resources
Source: JMIR Ment Health. 2019 Apr 5;6(4):e11686. doi: 10.2196/11686 (PMC6473206; doi:10.2196/11686)

Image 1. CTBH website homepage.

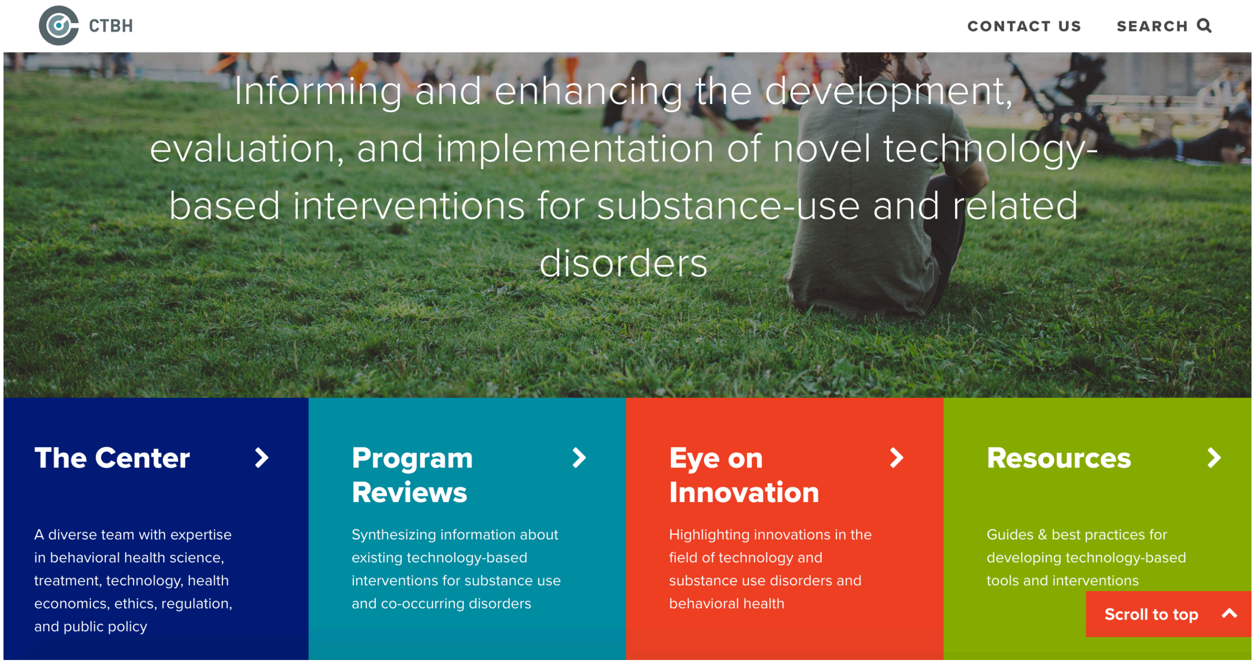

Image 2. Program Reviews filtered for substance use disorders.

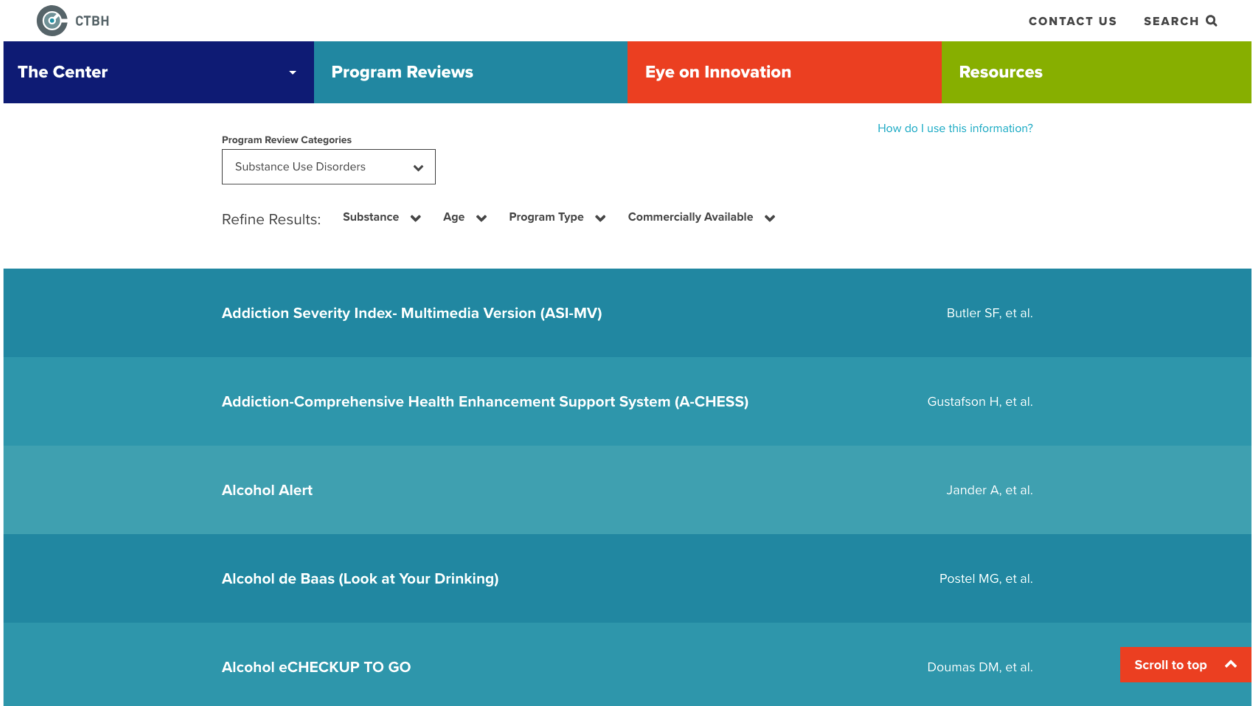

Image 3. Eye on Innovation landing page.

CTBH

CONTACT US   SEARCH

The Center | Program Reviews | Eye on Innovation | Resources

EYE ON INNOVATION

07/20/2018

Influence of flavors on the propagation of e-cigarette-related information: Social media study.

Zhou J, Zhang Q, Zeng DD, Tsui KL. (2018). Influence of flavors on the propagation of e-cigarette-related information: Social media study. JMIR Public Health and Surveillance. 4(1):e27. doi: 10.2196/publichealth.7998

Researchers analyzed user interactions on e-cigarette related Facebook pages to evaluate how flavors affect propagation of e-cigarette-related posts on social media. Read More

Share:

Posted in: Cutting Edge Literature // Tagged: big data; e-cigarettes; social media

Tags

- mhealth (327)
- mobile apps (263)
- mental health (142)
- substance use (131)
- policy and regulation (108)
- treatment (104)
- digital health (104)
- internet-based (98)

Image 4. Resources landing page.

CTBH

CONTACT US   SEARCH

The Center | Program Reviews | Eye on Innovation | Resources

RESOURCE CATEGORIES

- Measures for Implementation Studies
- Roadmap for Implementation of mHealth
- Science of Behavior Change (SOBC) Measures Repository
- Social Media Recruitment Methods

Coming Soon:

Standard Operating Procedures for Technology Research Trials

Sample Consent Forms

Developments in Data Security

Scroll to top

Image 5. CTBH Twitter account.

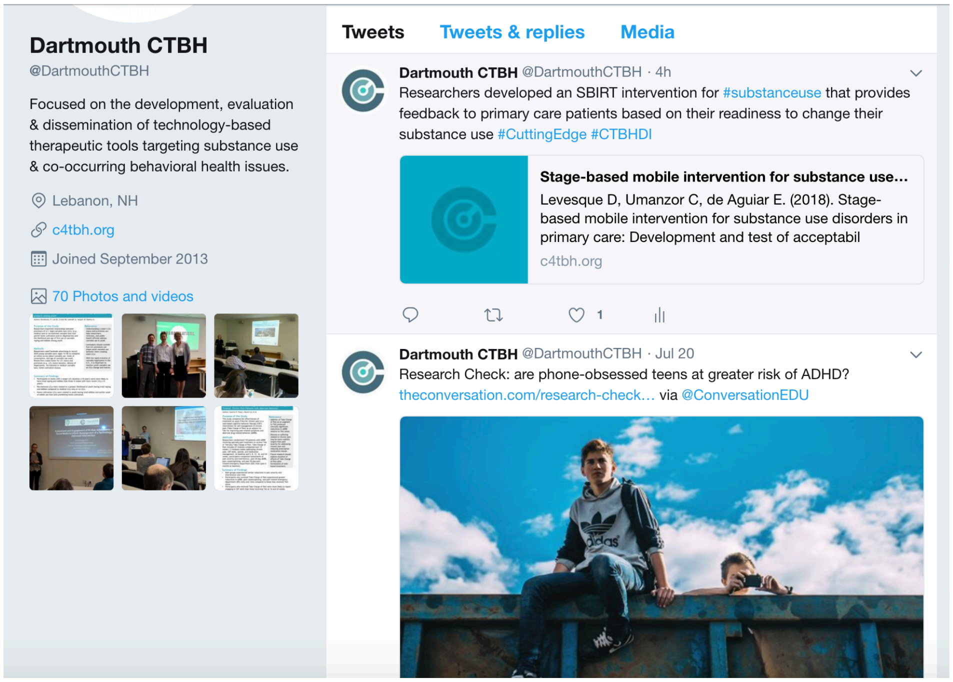

Image 6. CTBH Facebook page.

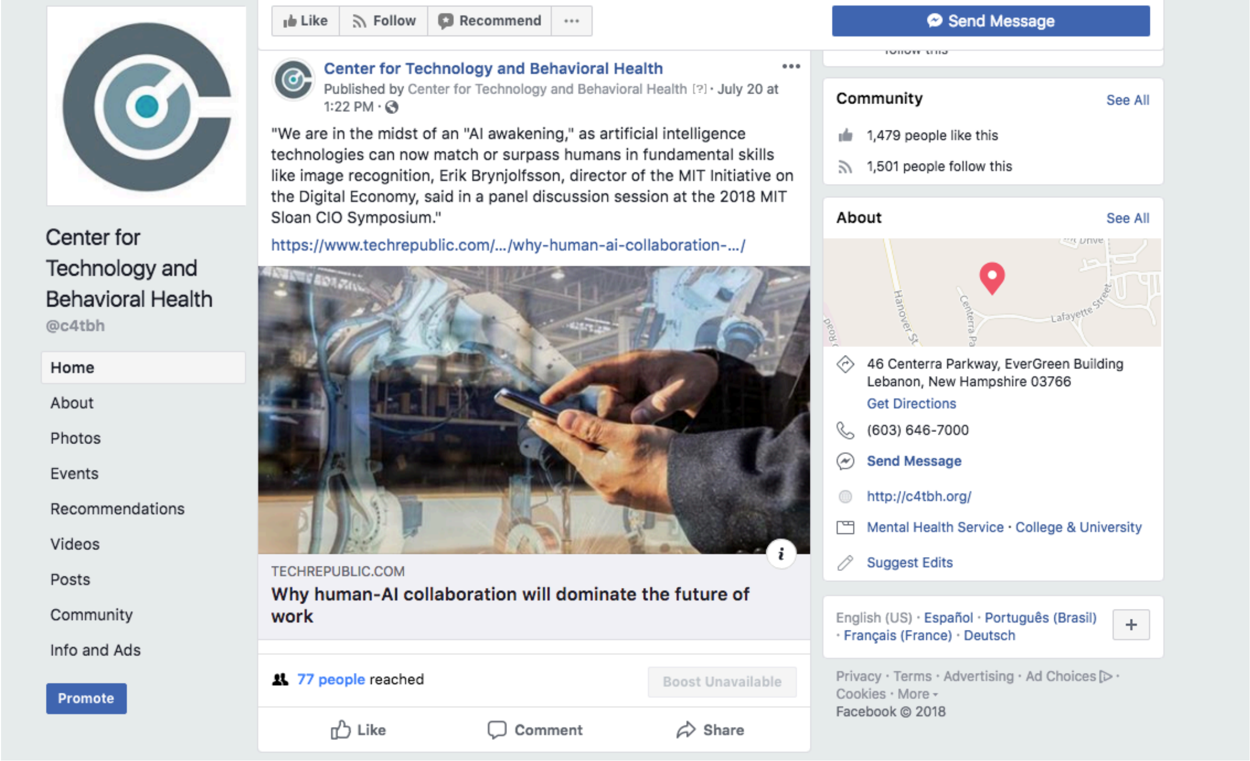

Supplement: Multimedia Appendix 1 [file mental_v6i4e11686_app1.pdf]
